# Corporate Policy, Male Breadwinners, and Their Family Care in Aging Japan

**DOI:** 10.1093/geroni/igab046.1889

**Published:** 2021-12-17

**Authors:** Hiroko Costantini, Glenda Roberts

**Affiliations:** 1 SciencesPo, Paris, Ile-de-France, France; 2 Waseda University, Tokyo, Tokyo, Japan

## Abstract

A rapidly emerging set of carers are men who combine care for older relatives with employment. In Japan, a 2015 government initiative aimed at reforming work to make employment and care compatible by 2020 failed to reduce the approximately 100,000 annually quitting employment mainly due to care for older relatives. This paper aims to evaluate the initiative’s limited impact through a multilevel understanding of the roll-out of the family care policy. Stakeholder views, based on 32 interviews including with employers, the Japanese Business Federation, local care providers and NPOs, are juxtaposed with the perspectives of employed male family carers drawn from 37 qualitative in-depth narrative interviews complemented by participant observation in the Tokyo area in 2019. The ethnographic fieldwork evidences informants’ diverse engagement with care for older relatives underpinned by strongly held cultural views of care provision being a ‘private’ issue, which contrasts with government attempts to make family care a ‘social’ issue by broadening stakeholder participation. Further, corporates tend to have tacit reluctance to transform working practices to accommodate care. Thus, employed men’s devotion to work competes with the culturally embedded notion that carers should be committed to care provision. In conclusion, such a disjuncture is a major factor in the government initiative’s failure. Although cultural values and meanings in policy evaluation theories are often neglected, this research points to the significance of ongoing (re)construction of the socio-cultural notions congruent with social policy enablement.

